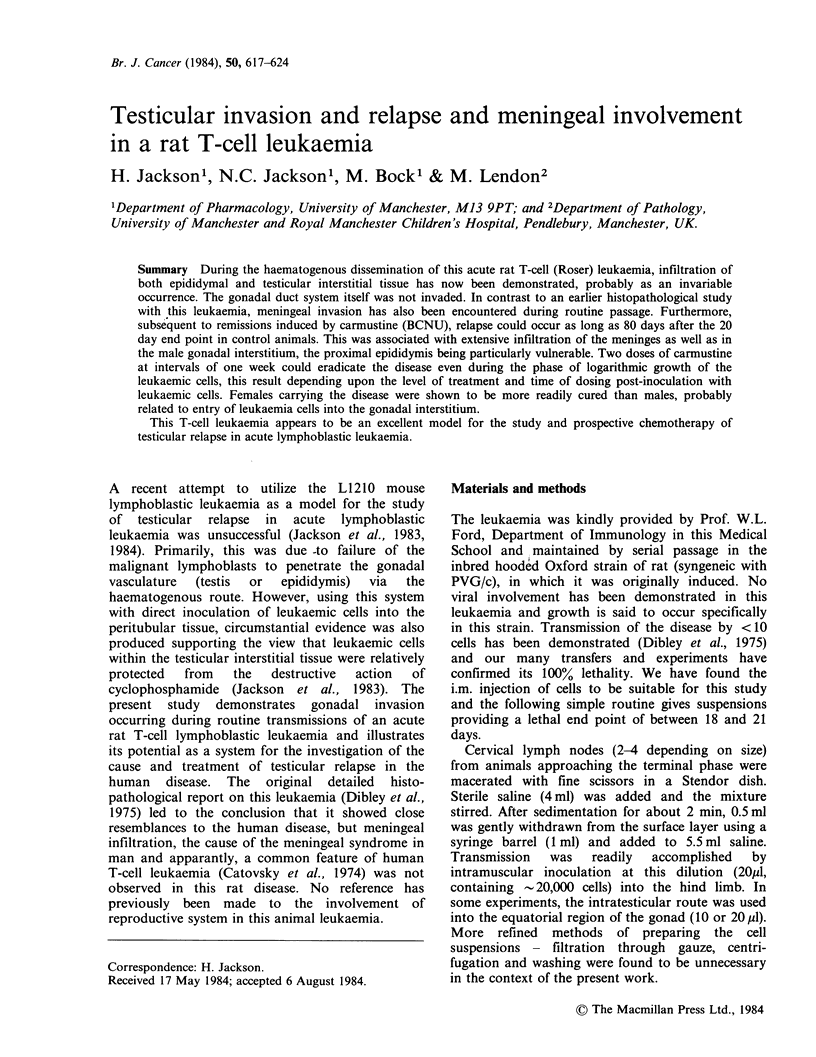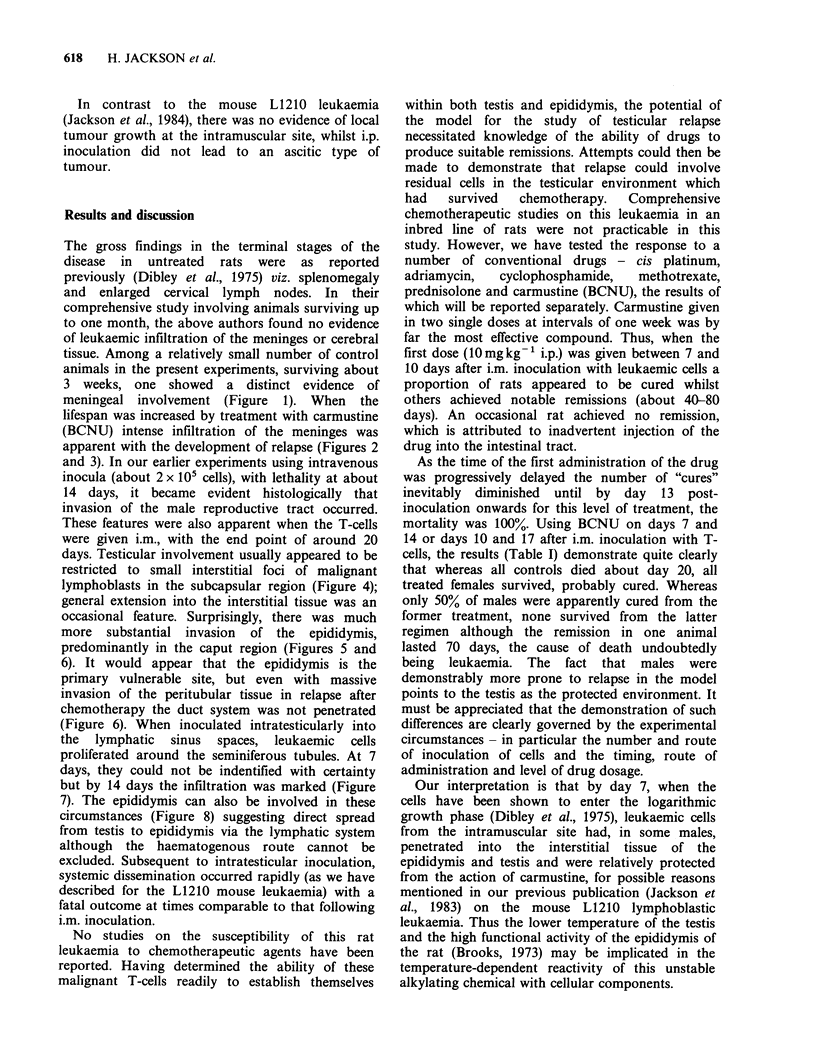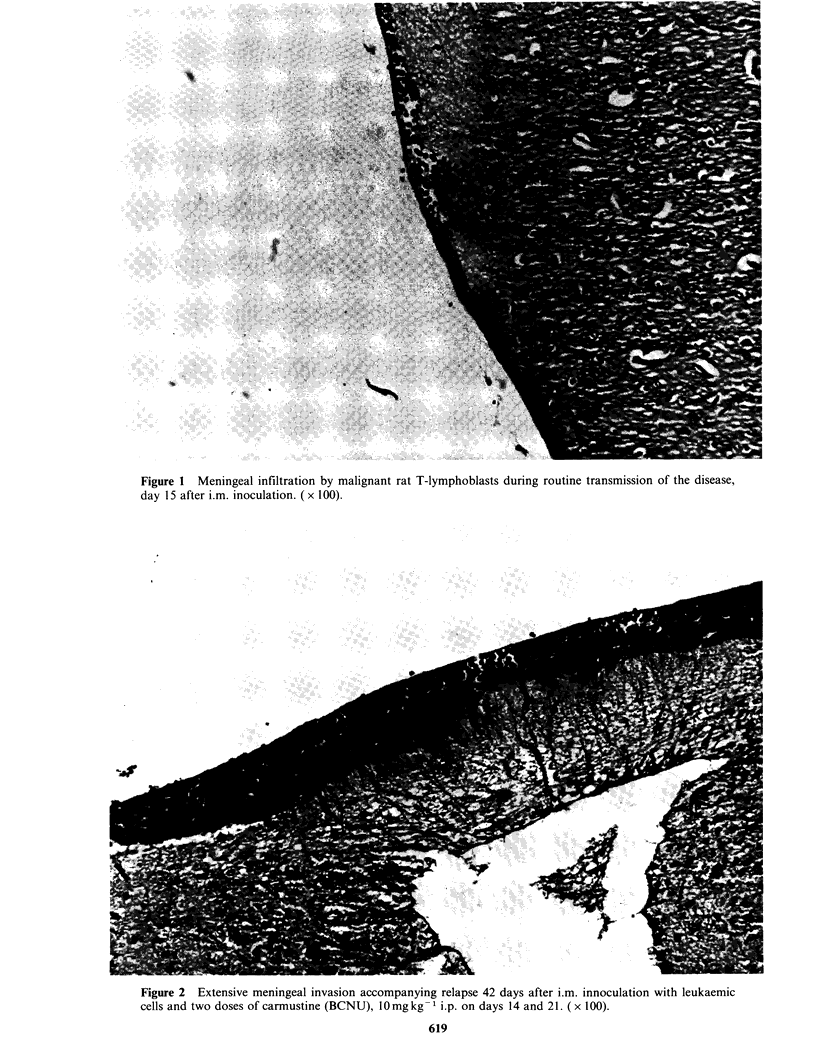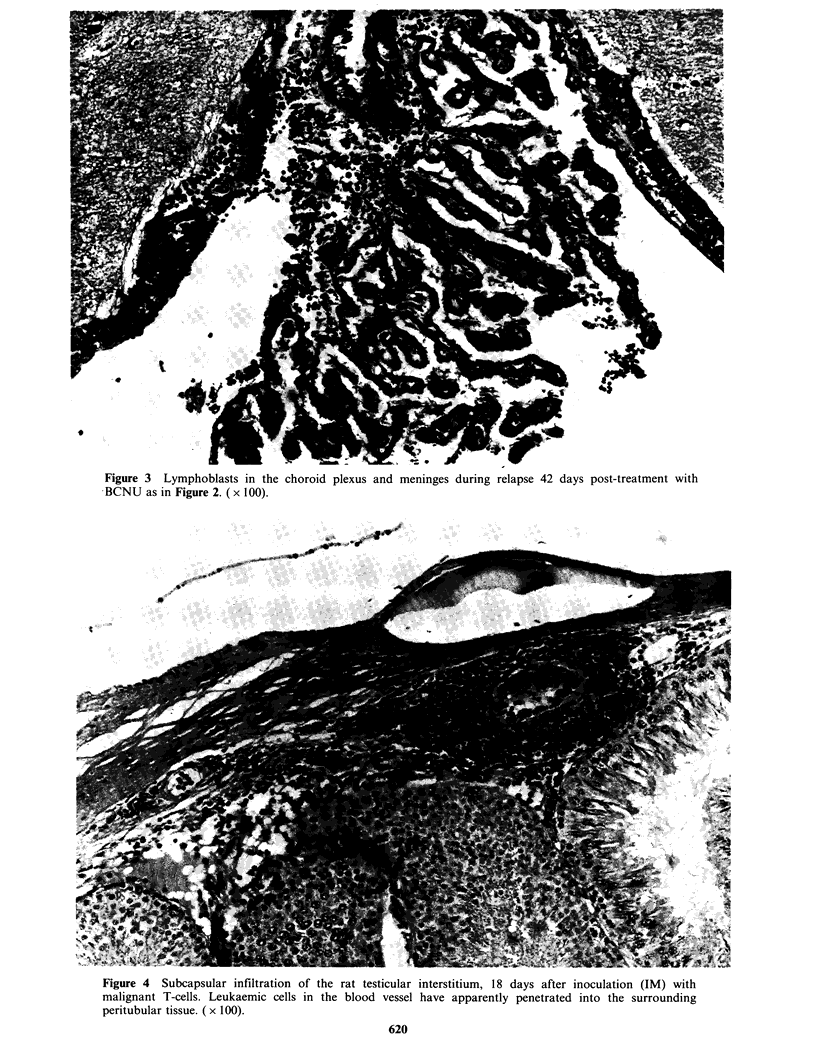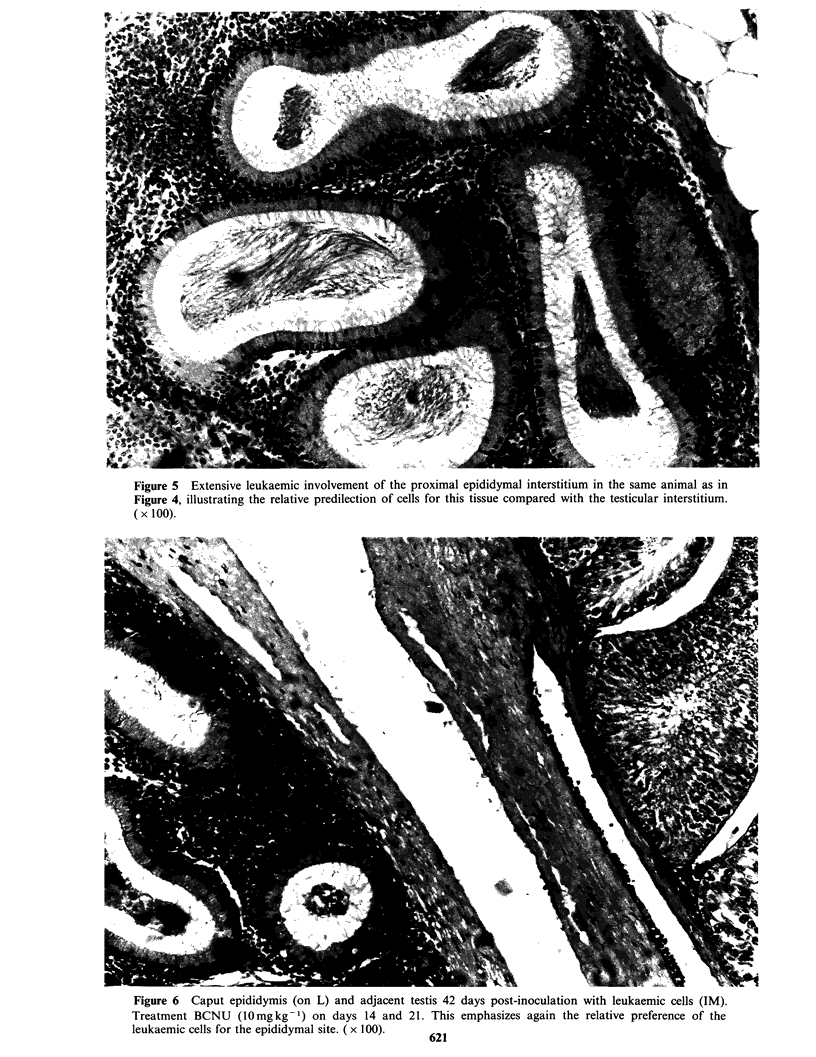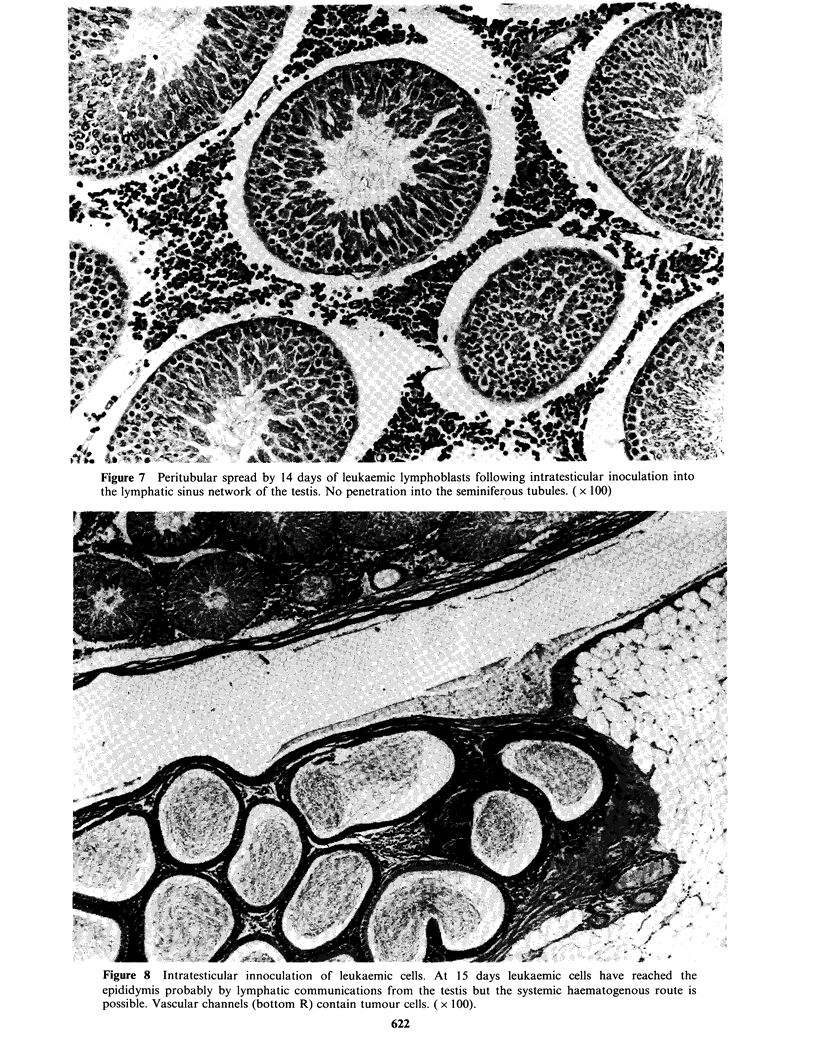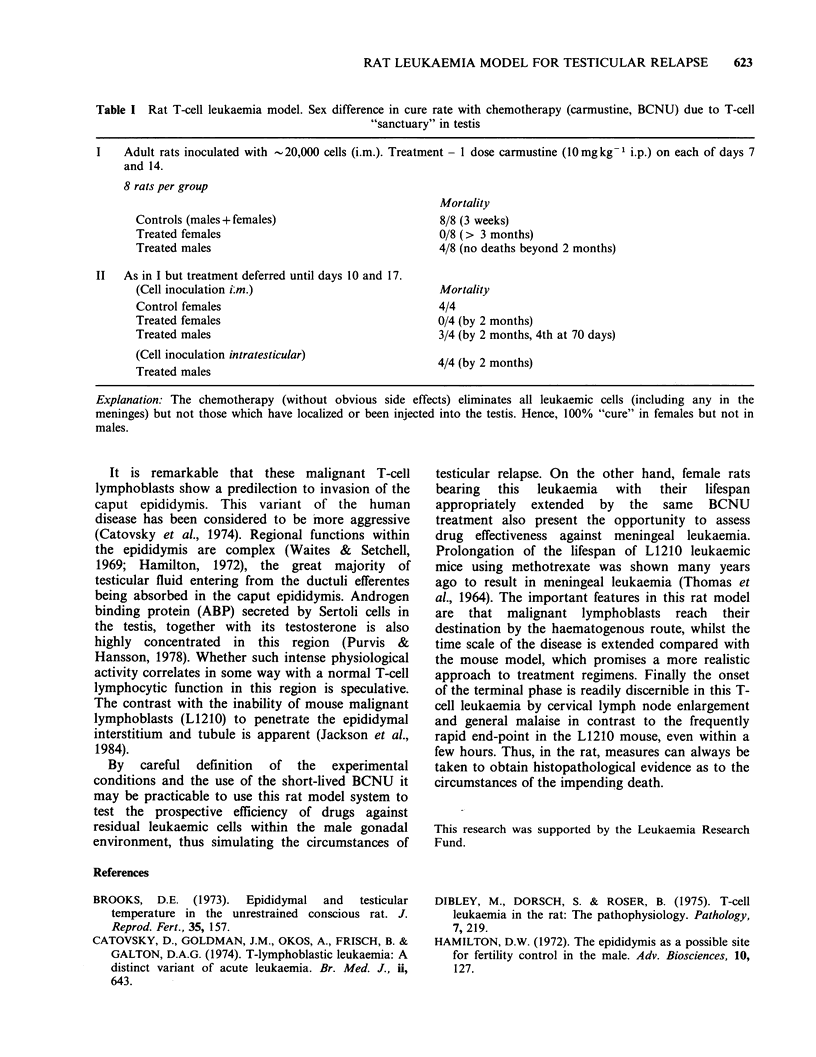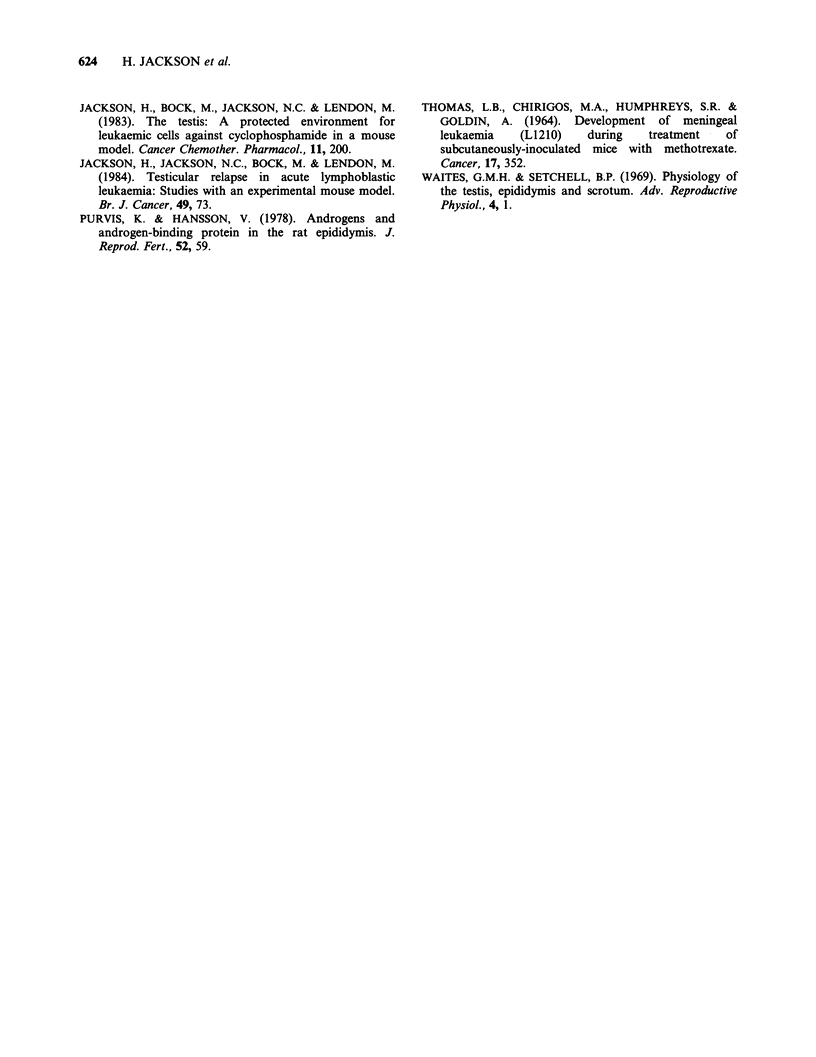# Testicular invasion and relapse and meningeal involvement in a rat T-cell leukaemia.

**DOI:** 10.1038/bjc.1984.228

**Published:** 1984-11

**Authors:** H. Jackson, N. C. Jackson, M. Bock, M. Lendon

## Abstract

**Images:**


*Br. J. Cancer* (1984), **50**, 617–624

### Testicular invasion and relapse and meningeal involvement in a rat T-cell leukaemia

H. Jackson[1], N.C. Jackson[1], M. Bock[1] & M. Lendon[2]

[1]*Department of Pharmacology, University of Manchester, M13 9PT; and* [2]*Department of Pathology, University of Manchester and Royal Manchester Children's Hospital, Pendlebury, Manchester, UK.*

**Summary** During the haematogenous dissemination of this acute rat T-cell (Roser) leukaemia, infiltration of both epididymal and testicular interstitial tissue has now been demonstrated, probably as an invariable occurrence. The gonadal duct system itself was not invaded. In contrast to an earlier histopathological study with this leukaemia, meningeal invasion has also been encountered during routine passage. Furthermore, subsequent to remissions induced by carmustine (BCNU), relapse could occur as long as 80 days after the 20 day end point in control animals. This was associated with extensive infiltration of the meninges as well as in the male gonadal interstitium, the proximal epididymis being particularly vulnerable. Two doses of carmustine at intervals of one week could eradicate the disease even during the phase of logarithmic growth of the leukaemic cells, this result depending upon the level of treatment and time of dosing post-inoculation with leukaemic cells. Females carrying the disease were shown to be more readily cured than males, probably related to entry of leukaemia cells into the gonadal interstitium.

This T-cell leukaemia appears to be an excellent model for the study and prospective chemotherapy of testicular relapse in acute lymphoblastic leukaemia.

A recent attempt to utilize the L1210 mouse lymphoblastic leukaemia as a model for the study of testicular relapse in acute lymphoblastic leukaemia was unsuccessful (Jackson *et al.*, 1983, 1984). Primarily, this was due to failure of the malignant lymphoblasts to penetrate the gonadal vasculature (testis or epididymis) via the haematogenous route. However, using this system with direct inoculation of leukaemic cells into the peritubular tissue, circumstantial evidence was also produced supporting the view that leukaemic cells within the testicular interstitial tissue were relatively protected from the destructive action of cyclophosphamide (Jackson *et al.*, 1983). The present study demonstrates gonadal invasion occurring during routine transmissions of an acute rat T-cell lymphoblastic leukaemia and illustrates its potential as a system for the investigation of the cause and treatment of testicular relapse in the human disease. The original detailed histopathological report on this leukaemia (Dibley *et al.*, 1975) led to the conclusion that it showed close resemblances to the human disease, but meningeal infiltration, the cause of the meningeal syndrome in man and apparently, a common feature of human T-cell leukaemia (Catovsky *et al.*, 1974) was not observed in this rat disease. No reference has previously been made to the involvement of reproductive system in this animal leukaemia.

Correspondence: H. Jackson.
Received 17 May 1984; accepted 6 August 1984.

### Materials and methods

The leukaemia was kindly provided by Prof. W.L. Ford, Department of Immunology in this Medical School and maintained by serial passage in the inbred hooded Oxford strain of rat (syngeneic with PVG/c), in which it was originally induced. No viral involvement has been demonstrated in this leukaemia and growth is said to occur specifically in this strain. Transmission of the disease by <10 cells has been demonstrated (Dibley *et al.*, 1975) and our many transfers and experiments have confirmed its 100% lethality. We have found the i.m. injection of cells to be suitable for this study and the following simple routine gives suspensions providing a lethal end point of between 18 and 21 days.

Cervical lymph nodes (2–4 depending on size) from animals approaching the terminal phase were macerated with fine scissors in a Stendor dish. Sterile saline (4 ml) was added and the mixture stirred. After sedimentation for about 2 min, 0.5 ml was gently withdrawn from the surface layer using a syringe barrel (1 ml) and added to 5.5 ml saline. Transmission was readily accomplished by intramuscular inoculation at this dilution (20 $\mu$l, containing ~20,000 cells) into the hind limb. In some experiments, the intratesticular route was used into the equatorial region of the gonad (10 or 20 $\mu$l). More refined methods of preparing the cell suspensions – filtration through gauze, centrifugation and washing were found to be unnecessary in the context of the present work.

© The Macmillan Press Ltd., 1984

In contrast to the mouse L1210 leukaemia (Jackson *et al.*, 1984), there was no evidence of local tumour growth at the intramuscular site, whilst i.p. inoculation did not lead to an ascitic type of tumour.

### Results and discussion

The gross findings in the terminal stages of the disease in untreated rats were as reported previously (Dibley *et al.*, 1975) *viz.* splenomegaly and enlarged cervical lymph nodes. In their comprehensive study involving animals surviving up to one month, the above authors found no evidence of leukaemic infiltration of the meninges or cerebral tissue. Among a relatively small number of control animals in the present experiments, surviving about 3 weeks, one showed a distinct evidence of meningeal involvement (Figure 1). When the lifespan was increased by treatment with carmustine (BCNU) intense infiltration of the meninges was apparent with the development of relapse (Figures 2 and 3). In our earlier experiments using intravenous inocula (about $2 \times 10^5$ cells), with lethality at about 14 days, it became evident histologically that invasion of the male reproductive tract occurred. These features were also apparent when the T-cells were given i.m., with the end point of around 20 days. Testicular involvement usually appeared to be restricted to small interstitial foci of malignant lymphoblasts in the subcapsular region (Figure 4); general extension into the interstitial tissue was an occasional feature. Surprisingly, there was much more substantial invasion of the epididymis, predominantly in the caput region (Figures 5 and 6). It would appear that the epididymis is the primary vulnerable site, but even with massive invasion of the peritubular tissue in relapse after chemotherapy the duct system was not penetrated (Figure 6). When inoculated intratesticularly into the lymphatic sinus spaces, leukaemic cells proliferated around the seminiferous tubules. At 7 days, they could not be indentified with certainty but by 14 days the infiltration was marked (Figure 7). The epididymis can also be involved in these circumstances (Figure 8) suggesting direct spread from testis to epididymis via the lymphatic system although the haematogenous route cannot be excluded. Subsequent to intratesticular inoculation, systemic dissemination occurred rapidly (as we have described for the L1210 mouse leukaemia) with a fatal outcome at times comparable to that following i.m. inoculation.

No studies on the susceptibility of this rat leukaemia to chemotherapeutic agents have been reported. Having determined the ability of these malignant T-cells readily to establish themselves within both testis and epididymis, the potential of the model for the study of testicular relapse necessitated knowledge of the ability of drugs to produce suitable remissions. Attempts could then be made to demonstrate that relapse could involve residual cells in the testicular environment which had survived chemotherapy. Comprehensive chemotherapeutic studies on this leukaemia in an inbred line of rats were not practicable in this study. However, we have tested the response to a number of conventional drugs – *cis* platinum, adriamycin, cyclophosphamide, methotrexate, prednisolone and carmustine (BCNU), the results of which will be reported separately. Carmustine given in two single doses at intervals of one week was by far the most effective compound. Thus, when the first dose ($10\,mg\,kg^{-1}$ i.p.) was given between 7 and 10 days after i.m. inoculation with leukaemic cells a proportion of rats appeared to be cured whilst others achieved notable remissions (about 40–80 days). An occasional rat achieved no remission, which is attributed to inadvertent injection of the drug into the intestinal tract.

As the time of the first administration of the drug was progressively delayed the number of "cures" inevitably diminished until by day 13 post-inoculation onwards for this level of treatment, the mortality was 100%. Using BCNU on days 7 and 14 or days 10 and 17 after i.m. inoculation with T-cells, the results (Table I) demonstrate quite clearly that whereas all controls died about day 20, all treated females survived, probably cured. Whereas only 50% of males were apparently cured from the former treatment, none survived from the latter regimen although the remission in one animal lasted 70 days, the cause of death undoubtedly being leukaemia. The fact that males were demonstrably more prone to relapse in the model points to the testis as the protected environment. It must be appreciated that the demonstration of such differences are clearly governed by the experimental circumstances – in particular the number and route of inoculation of cells and the timing, route of administration and level of drug dosage.

Our interpretation is that by day 7, when the cells have been shown to enter the logarithmic growth phase (Dibley *et al.*, 1975), leukaemic cells from the intramuscular site had, in some males, penetrated into the interstitial tissue of the epididymis and testis and were relatively protected from the action of carmustine, for possible reasons mentioned in our previous publication (Jackson *et al.*, 1983) on the mouse L1210 lymphoblastic leukaemia. Thus the lower temperature of the testis and the high functional activity of the epididymis of the rat (Brooks, 1973) may be implicated in the temperature-dependent reactivity of this unstable alkylating chemical with cellular components.

**Figure 1** Meningeal infiltration by malignant rat T-lymphoblasts during routine transmission of the disease, day 15 after i.m. inoculation. ( × 100).

**Figure 2** Extensive meningeal invasion accompanying relapse 42 days after i.m. innoculation with leukaemic cells and two doses of carmustine (BCNU), $10\,mg\,kg^{-1}$ i.p. on days 14 and 21. ( × 100).

**Figure 3** Lymphoblasts in the choroid plexus and meninges during relapse 42 days post-treatment with BCNU as in **Figure 2**. ( × 100).

**Figure 4** Subcapsular infiltration of the rat testicular interstitium, 18 days after inoculation (IM) with malignant T-cells. Leukaemic cells in the blood vessel have apparently penetrated into the surrounding peritubular tissue. ( × 100).

**Figure 5** Extensive leukaemic involvement of the proximal epididymal interstitium in the same animal as in **Figure 4**, illustrating the relative predilection of cells for this tissue compared with the testicular interstitium. (×100).

**Figure 6** Caput epididymis (on L) and adjacent testis 42 days post-inoculation with leukaemic cells (IM). Treatment BCNU ($10\,mg\,kg^{-1}$) on days 14 and 21. This emphasizes again the relative preference of the leukaemic cells for the epididymal site. (×100).

**Figure 7** Peritubular spread by 14 days of leukaemic lymphoblasts following intratesticular inoculation into the lymphatic sinus network of the testis. No penetration into the seminiferous tubules. ( × 100)

**Figure 8** Intratesticular innoculation of leukaemic cells. At 15 days leukaemic cells have reached the epididymis probably by lymphatic communications from the testis but the systemic haematogenous route is possible. Vascular channels (bottom R) contain tumour cells. ( × 100).

**Table I** Rat T-cell leukaemia model. Sex difference in cure rate with chemotherapy (carmustine, BCNU) due to T-cell "sanctuary" in testis

| | | |
|---|---|---|
| I | Adult rats inoculated with ~20,000 cells (i.m.). Treatment – 1 dose carmustine ($10\,mg\,kg^{-1}$ i.p.) on each of days 7 and 14. | |
| | *8 rats per group* | |
| | | *Mortality* |
| | Controls (males+females) | 8/8 (3 weeks) |
| | Treated females | 0/8 (> 3 months) |
| | Treated males | 4/8 (no deaths beyond 2 months) |
| II | As in I but treatment deferred until days 10 and 17. | |
| | (Cell inoculation *i.m.*) | *Mortality* |
| | Control females | 4/4 |
| | Treated females | 0/4 (by 2 months) |
| | Treated males | 3/4 (by 2 months, 4th at 70 days) |
| | (Cell inoculation *intratesticular*) Treated males | 4/4 (by 2 months) |

*Explanation:* The chemotherapy (without obvious side effects) eliminates all leukaemic cells (including any in the meninges) but not those which have localized or been injected into the testis. Hence, 100% "cure" in females but not in males.

It is remarkable that these malignant T-cell lymphoblasts show a predilection to invasion of the caput epididymis. This variant of the human disease has been considered to be more aggressive (Catovsky *et al.*, 1974). Regional functions within the epididymis are complex (Waites & Setchell, 1969; Hamilton, 1972), the great majority of testicular fluid entering from the ductuli efferentes being absorbed in the caput epididymis. Androgen binding protein (ABP) secreted by Sertoli cells in the testis, together with its testosterone is also highly concentrated in this region (Purvis & Hansson, 1978). Whether such intense physiological activity correlates in some way with a normal T-cell lymphocytic function in this region is speculative. The contrast with the inability of mouse malignant lymphoblasts (L1210) to penetrate the epididymal interstitium and tubule is apparent (Jackson *et al.*, 1984).

By careful definition of the experimental conditions and the use of the short-lived BCNU it may be practicable to use this rat model system to test the prospective efficiency of drugs against residual leukaemic cells within the male gonadal environment, thus simulating the circumstances of testicular relapse. On the other hand, female rats bearing this leukaemia with their lifespan appropriately extended by the same BCNU treatment also present the opportunity to assess drug effectiveness against meningeal leukaemia. Prolongation of the lifespan of L1210 leukaemic mice using methotrexate was shown many years ago to result in meningeal leukaemia (Thomas *et al.*, 1964). The important features in this rat model are that malignant lymphoblasts reach their destination by the haematogenous route, whilst the time scale of the disease is extended compared with the mouse model, which promises a more realistic approach to treatment regimens. Finally the onset of the terminal phase is readily discernible in this T-cell leukaemia by cervical lymph node enlargement and general malaise in contrast to the frequently rapid end-point in the L1210 mouse, even within a few hours. Thus, in the rat, measures can always be taken to obtain histopathological evidence as to the circumstances of the impending death.

This research was supported by the Leukaemia Research Fund.

### References

BROOKS, D.E. (1973). Epididymal and testicular temperature in the unrestrained conscious rat. *J. Reprod. Fert.*, **35**, 157.

CATOVSKY, D., GOLDMAN, J.M., OKOS, A., FRISCH, B. & GALTON, D.A.G. (1974). T-lymphoblastic leukaemia: A distinct variant of acute leukaemia. *Br. Med. J.*, **ii**, 643.

DIBLEY, M., DORSCH, S. & ROSER, B. (1975). T-cell leukaemia in the rat: The pathophysiology. *Pathology*, **7**, 219.

HAMILTON, D.W. (1972). The epididymis as a possible site for fertility control in the male. *Adv. Biosciences*, **10**, 127.

JACKSON, H., BOCK, M., JACKSON, N.C. & LENDON, M. (1983). The testis: A protected environment for leukaemic cells against cyclophosphamide in a mouse model. *Cancer Chemother. Pharmacol.*, **11**, 200.

JACKSON, H., JACKSON, N.C., BOCK, M. & LENDON, M. (1984). Testicular relapse in acute lymphoblastic leukaemia: Studies with an experimental mouse model. *Br. J. Cancer*, **49**, 73.

PURVIS, K. & HANSSON, V. (1978). Androgens and androgen-binding protein in the rat epididymis. *J. Reprod. Fert.*, **52**, 59.

THOMAS, L.B., CHIRIGOS, M.A., HUMPHREYS, S.R. & GOLDIN, A. (1964). Development of meningeal leukaemia (L1210) during treatment of subcutaneously-inoculated mice with methotrexate. *Cancer*, **17**, 352.

WAITES, G.M.H. & SETCHELL, B.P. (1969). Physiology of the testis, epididymis and scrotum. *Adv. Reproductive Physiol.*, **4**, 1.